# Hospital Expenditure.—The Commissariat.—I

**Published:** 1895-10-05

**Authors:** 


### The Institutional Workshop.

### HOSPITAL EXPENDITURE.—THE COMMISSARIAT.

### I.—SPECIAL FEATURES OF THE HOSPITAL HOUSEHOLD.

The domestic economy of a household is governed by four main considerations: the number of persons to be fed; their description; the food supply; and the system of management. Under each of these heads the hospital presents distinct peculiarities and labours under impediments to economy from which the ordinary household, even the large school or kindred institution, is to a great extent free.

Large numbers, although popularly considered economical, do in themselves constitute certain well understood difficulties in the commissariat, and varying numbers do so to a still greater extent. Where the number of the population is both large and fluctuating, as is the case in many hospitals, the difficulty of providing exactly enough and no more is enormously increased, and when it is remembered that these variations in number extend to each ward which differs almost daily in its requirements, it is seen that a strain will rest upon even the most closely devised system, if waste is to be altogether avoided.

But a large hospital comprises within its walls a population not only as large, but as varied in its requirements as that of a small town. The inhabitants differ from each other in every possible particular, and the individual distinctions are nowhere more apparent than in diet. To begin with the patients. How can the sick child on a pint of milk a day be reckoned with the convalescent navvy who is building up his strength on chops and porter and everything nourishing he can get? Yet both are summarised at the same amount per head in the yearly average. Even if the hospital household consisted of patients alone, the average obtained by dividing the food consumed among the beds would be necessarily rough, and comparisons might be misleading. In hospitals like the Poplar for Accidents, and the Seamen's, for example, where the inmates consist chiefly of men under surgical treatment, this difference in diet must be allowed its full weight, since the ratio of patients on full diet is a more important factor than is generally supposed in estimating the cost of provisions.

But the patients are only a section of the household, and as will be shown, scarcely the most important section from the housekeeper's point of view. There is the doctors' table to be supplied, and it is fitting that these gentlemen, whose salary is nominal or nil and whose work is exceptionally arduous, should be catered for well and liberally. A second table is commonly provided for members of the household, dispensers, steward, &c., who receive board; in some instances these receive partial board only, lunch and tea or lunch on certain days, while in many hospitals members of the committee and casual visitors are liable to drop in to meals, and make the housekeeper's reckoning of heads a matter of increased uncertainty. The nurses' table is, perhaps, the most uniform and easily estimated item in the whole household, but the variety of meals for day and night nurses introduces here also an element peculiar to the hospital household, and not without danger to the economist.

The servants' table will generally be divided into that for the porters, engineer, &c., and that for the female servants, both complicated again by the feature of partial board. Lastly, comes the unrecognised army of skirmishers known as scrubbers, in many hospitals the licensed recipients of "remains," and in very few entirely cut off from kitchen benefits.

When all these points are taken into consideration and the fact realised that in each particular every hospital has its own custom, is it any wonder that they should be found to differ largely in the amount expended on provisions, and that the average per bed, unexplained by household arrangements, should often have proved entirely fallacious as a test of true economy?

In regard to the food supply, the hospital is again somewhat exceptionally situated. Side by side with the struggle for economy is the mandate on the part of the public that all shall be "of the best," and the necessity for supplying more or less expensive "extras" to certain patients at the order of the doctor in attendance. Such extras must be supplied irrespective of season, with the possible result of raising the contract price for the whole year to cover the loss inflicted in scarce months. Nominally the patients, as recipients of charitable aid, are fed merely on good and simple food; practically, it is found impossible to reduce a sick man's fancies to rigid discipline, and hospitals like the Middlesex, receiving large numbers of bad medical cases, are forced to study the appetites of their patients to an extent which directly affects the average expenditure.

But it is in respect of management that the hospital is very frequently at its greatest disadvantage as compared with the ordinary household. The essence of good housekeeping consists in the control of a central authority, responsible for the quality and price of the provisions; responsible also for the amount consumed by the household. In too many institutions this central authority is subdivided until the sense of responsibility is to a great extent frittered away. Responsibility tends always to decline in force from the head downwards. It is highest in those who receive the accounts, and on whose shoulders the direct burden of the finance rests. It is lowest in those who actually dispense or receive the articles provided. The doctor orders what he sees the patient desires; the sister requires an ample supply for her ward; both laudably anxious that the patient shall be well treated The matron, again, responsible for the well-being of the nurses, feels bound to guard them from any cheeseparing policy; the steward must see that the doctors' table is properly provided, and that no complaints proceed from this quarter, while the cook is queen in her own domain, and takes very good care that it is not the servants who come short. The secretary presenting the accounts to the committee, and nominally responsible for all, finds himself, unless exceptionally far-seeing and well-informed, with very little real power of interference. Remonstrance is easily overborne by those in charge of each department, and urgent reasons are readily forthcoming for any increase in expenditure, while the smallest change in the direction of economy is commonly received as something akin to a personal insult. This does not, of course, apply to all hospitals. The management in a few cases resembles a nicely adjusted and well oiled piece of mechanism, all parts working together in admirable harmony at the least expenditure of friction, time, and money. But there can be no doubt that in many instances an awkward system of management, handed down from laxer times, constitutes a serious bar to the practice of true economy.

Nothing is easier than to fling stones at hospital extravagance. A correspondence on the lines of violent and ignorant accusation is getting to be one of the familiar newspaper stocks in trade for the silly season. Out of much senseless clamour one truth appears, however, to be slowly emerging, that popular criticism is the inevitable and salutary result of popular support, and this truth once clearly recognised by the hospital authorities will in the end break down all barriers of mystery and open the way to plain proofs of economy and efficiency.

